# Radiographic lumbosacral vertebral abnormalities and constipation in cats

**DOI:** 10.14202/vetworld.2021.492-498

**Published:** 2021-02-23

**Authors:** Chutimon Thanaboonnipat, Kamonchanok Kumjumroon, Kamonwan Boonkwang, Natthacha Tangsutthichai, Wassapon Sukserm, Nan Choisunirachon

**Affiliations:** 1 Department of Surgery, Faculty of Veterinary Science, Chulalongkorn University, Bangkok, 10330, Thailand; 2 Faculty of Veterinary Science, Chulalongkorn University, Bangkok, 10330, Thailand

**Keywords:** cat, constipation, large bowel, lumbosacral, vertebrae

## Abstract

**Background and Aim:**

Lumbosacral intervertebral disk disease (IVDD) in cats usually develops concurrent with constipation, spondylosis deformans, and sacralization. However, the prevalence of lumbar IVDD in cats was considered low, and there was less information on the incidence of non-traumatic lumbosacral vertebral abnormalities that may affect large bowel dysfunction. This study aimed to retrospectively investigate the relationship between non-traumatic lumbosacral vertebral abnormalities, both congenital and acquired, and large bowel dysfunction in cats.

**Materials and Methods:**

Of 3108 cats that were presented to the Diagnostic Imaging Unit, the Small Animal Teaching Hospital, Faculty of Veterinary Science, Chulalongkorn University, between March 2016 and February 2018, 1365 cats met the inclusion criteria. All abdominal radiographs were reviewed, and all subsequent data were recorded, including the presence of congenital or acquired lumbosacral lesions, number of lumbar vertebrae, and length of the second, fifth, and last lumbar vertebrae, including the type of lumbar abnormalities. Moreover, radiographic information relating to constipation and megacolon was also collected.

**Results:**

Non-traumatic lumbosacral vertebral abnormalities were observed in 29.74% of cats. The most common congenital lumbosacral vertebral abnormalities were six lumbar vertebrae, sacralization, and lumbarization, whereas most common acquired lumbosacral abnormalities were bone spur, narrowing disk space, spondylosis deformans, and lumbosacral degeneration, respectively. Cats with abnormal lumbosacral vertebrae are prone to have more problems with the large bowel (p=0.0057; odds ratio=1.731). Moreover, congenital and acquired lumbosacral abnormalities were also at risk of large bowel abnormalities (p=0.0069; odds ratio=1.920 and p<0.0001; odds ratio=4.107, respectively).

**Conclusion:**

This study revealed the evidence and distribution of the variation in feline lumbar anatomy and also elucidated that cats with abnormal lumbar vertebral columns were more likely to have problems with distal gastrointestinal tracts than those without.

## Introduction

In cats, constipation is a reversible loss of large bowel function caused by various problems, without predilection to age, sex, or breed [1,2]. The symptoms of constipation include difficulty in defecation, abnormal defecation, abdominal enlargement, tenesmus, infrequent defecation, and irregular fecal materials, such as small and round or ribbon-like feces. Affected cats may also have signs of dehydration, weight loss, abdominal pain, and mesenteric lymphadenopathy [3]. Inadequate consumption of fiber and water is believed to be one of the main risk factors. Moreover, some medications affect intestinal functions, for example, opioids, such as loperamide and diphenoxylate, and sedative drugs, such as xylazine [4]. In addition to constipation, obstipation usually occurs following intestinal obstructions, such as colorectal stricture [5], narrow pelvic canal from previous pelvic fracture and malunion [6,7], intestinal mass, and intrapelvic or sublumbar masses [6,8]. All causes lead to permanent loss of intestinal function [9], resulting in colonic dilation, due to the packed and hardened fecal materials, which can progress to hypertrophic megacolon, an irreversible condition of dramatically increased colon diameter [10]. In cases of idiopathic megacolon, most occurrences were observed in middle-aged cats and colonic smooth muscle dysfunction appeared to be involved in the pathogenesis, but the etiology is still unclear [11]. At present, many studies in both humans and cats are attempting to find medical treatment that effectively improves the abnormal signs of chronic constipation or megacolon, including defecation disorders [2,12,13].

Many environmental factors or other abnormalities, such as pelvic fracture, intra-abdominal mass, peritoneal effusion, dysautonomia, and abdominal foreign bodies, can increase the risk of large bowel dysfunctions [2,14-16]. Studies on the association between these problems and any of the non-traumatic or congenital vertebral abnormalities, including the incidence of latter abnormalities, are rare in cats, whereas they are well described in other species. In humans, either congenital or acquired spinal stenosis and bone spur may increase the risk of cauda equina syndrome (CES), subsequently causing atonic bowel, which develops to severe constipation [17]. In dogs, lumbosacral transitional vertebrae are believed to be one of the risk factors of fecal incontinence because of its association with CES [18]. Degenerative lumbosacral stenosis (DLSS), which causes fecal incontinence, is also commonly found in dogs. DLSS includes intervertebral disk disease (lumbosacral intervertebral disk disease [IVDD]), spondylolisthesis, and discospondylitis [19]. Despite several lumbosacral vertebral abnormalities in cats, such as hemivertebrae, transitional vertebrae, bone spurs, and spondylosis, the association of these abnormalities to CES in cats has not been previously reported [20]. The previous studies showed that lumbosacral IVDD was concurrently found with constipation [21] and spondylosis deformans and sacralization [22].

Since the prevalence of lumbar IVDD in cats was considered uncommon [23] and there was less information on the incidence of non-traumatic, lumbosacral vertebral abnormalities that may affect large bowel dysfunction. This study aimed to retrospectively investigate the incidence of non-traumatic lumbosacral vertebral abnormalities in cats and the relationships of lumbosacral vertebral abnormalities and constipation and/or megacolon in cats on abdominal radiographs.

## Materials and methods

### Ethical approval and informed consent

This study used clinical and radiographic information from the Small Animal Teaching Hospital, Faculty of Veterinary Science, Chulalongkorn University database only (including owned or unowned animals and data from retrospective studies). Established internationally recognized high standards of individual veterinary clinical patient care were followed. Ethical approval from a committee was, therefore, not necessarily required. Informed consent (either verbal or written) was obtained from the owner or legal custodian of all animals described in this work for the procedures undertaken.

### Study location and period

This study was designed as a retrospective study using patient information (both clinical and radiographic data) of cats that were presented to the Diagnostic Imaging Unit, the Small Animal Teaching Hospital, Faculty of Veterinary Science, Chulalongkorn University, in March 2016 and February 2018.

### Animals and experimental design

The data of the cats were considered and included if they revealed complete signalment and there were at least two orthogonal radiographic views of the whole abdomen that covered the region of interest (ROI) from the caudal thoracic vertebrae to ischial tuberosity. Cases of incomplete clinical information, uncovering ROI of abdominal radiograph than the former information, evidence of previous traumatic injuries from abdominal radiographs, pregnancy, skeletal immaturity, and other conditions that might cause colorectal obstruction or constipation, such as intra-abdominal mass, were excluded from the study. Then, all clinical information were collected.

### Radiographic analysis

#### Characterization of lumbosacral vertebrae

Abdominal radiographs of all included cats were collected as digital information and communication system (DICOM) files and were reviewed using the DICOM viewer software (OsiriX, Switzerland). First, all abdominal radiographs were reviewed and categorized into the normal group (Group 1; Gr.1) or the non-traumatic lumbosacral vertebral abnormality group (Group 2; Gr. 2). Briefly, abdominal radiographs of the cats were considered abnormal if they presented either congenital lesions (Group 2A; Gr. 2A), such as less or excessive lumbar vertebral number, hemivertebrae, lumbarization, and sacralization, or non-traumatic acquired lesions (Group 2B; Gr. 2B) of spondylosis deformans, ventral bone spur, narrowing disk space, and lumbosacral degeneration. In the case of a single presentation of lumbosacral abnormalities, the lesion was categorized to be the cranial area if the lesion was located at the area from the first to the third lumbar vertebrae or caudal area if the lesion was located at the area from the fourth lumbar vertebrae to the first sacrum.

#### Characterization of large bowel dysfunction

**Figure-1:**
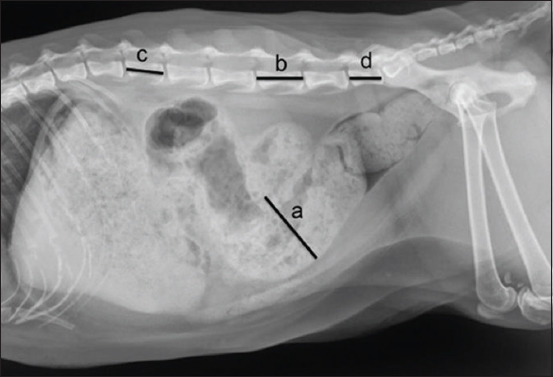
Lateral abdominal radiograph of a cat indicating the measurement method for large bowel dilatation. The maximal diameter of the colon (a) was measured and compared to the length of the fifth lumbar vertebrae (b). In addition, the length of the second (c) and the seventh lumbar vertebrae (d) was measured and compared between normal cats.

The lateral abdominal radiograph of each cat was observed for gastrointestinal (GI) tract appearance, including characteristics of the large bowel contents. The colon diameter was recorded at the most dilated portion (MD). Then, colon diameters were compared to the length of the fifth lumbar (L5) vertebrae. In addition to the length of L5, the length of the second (L2) and last (LL) lumbar vertebrae was measured and compared (Figure-1). All measurements were performed using digital calipers, and all parameters were recorded. The cutoff values were applied to differentiate the types of large bowel dysfunction, followed previous information [9]. Briefly, the colon that contained large bowel contents and had an MD diameter 1.28 times larger than the L5 length was considered to have constipation, whereas the colon that contained fecal contents and had an MD diameter 1.48 times larger than the L5 length was classified as megacolon [9].

### Statistical analysis

The descriptive data of clinical information were expressed as mean and standard error of the mean. Before the analysis of statistical significance, all data in each category were tested for normalization using the Kolmogorov–Smirnov test, and non-parametric Wilcoxon signed-rank test was applied. Relationships of lumbar vertebral abnormalities and any of the large bowel dysfunctions were tested by Fisher’s exact test. All data were analyzed using the GraphPad Prism 8^®^ software (GraphPad Software, CA, USA). The difference in parameters between groups was recognized as statistically significant if p<0.05 was considered.

## Results

### Animals

Of the 3108 cats, only 1365 cats were included in this study. The five most common breeds were domestic shorthair (n=1,074, 78.68%), Persian (n=155, 11.36%), mixed breed (n=40, 2.93%), Thai Wichianmas (n=30, 2.20%), and Scottish fold (n=23, 1.68%), respectively. The average age was 53.71±1.04 years, and the average body weight was 4.02±0.03 kg. There were 781 male cats (357 castrated and 424 intact cats) and 584 female cats (315 spayed and 269 intact cats). All clinical information, such as age, body weight, and sex of cats in each group, are summarized in Table-1.

**Table-1:** Clinical information such as number, sex, age (mean±SEM; months), and body weight (mean±SEM; kg) of the cats, all of the overall, normal lumbar sacral vertebrae, and non traumatic lumbar sacral lumbar abnormalities.

Clinical information	Gonadal status						

	Overall	Male	Intact male	Castrated male	Female	Intact female	Spayed female
Overall cats							
Number	1365	781	424	357	584	269	315
Age (months)	53.71±1.40	45.93±1.53	33.14±1.60	61.11±2.53	64.11±2.48	45.26±3.17	80.20±3.48
Body weight (kg)	4.02±0.03	4.40±0.04	4.07±0.05	4.78±0.06	3.50±0.04	3.27±0.06	3.70±0.06
Normal vertebrae							
Number	959	557	306	251	402	201	201
Age (months)	47.13±1.43	42.32±1.64	31.32±1.75	55.73±2.71	53.80±2.51	40.05±3.10	67.55±3.70
Body weight (kg)	4.01±0.04	4.39±0.05	4.02±0.06	4.85±0.08	3.48±0.05	3.25±0.07	3.70±0.07
Abnormal vertebrae							
Number	406	224	118	106	182	68	114
Age (months)	69.23±3.13	54.90±3.36	37.86±3.49	73.87±5.39	86.86±5.35	60.63±8.3	102.51±6.56
Body weight (kg)	4.03±0.06	4.41±0.08	4.21±0.11	4.64±0.11	3.57±0.08	3.33±0.12	3.71±0.10
Congenital							
Number	226	140	81	59	86	35	51
Age (months)	50.77±3.20	41.44±2.83	28.15±2.74	59.68±4.62	65.98±6.74	51.74±10.60	75.75±8.56
Body weight (kg)	4.06±0.08	4.35±0.10	4.10±0.13	4.69±0.15	3.58±0.11	3.33±0.16	3.76±0.14
Acquired							
Number	180	84	37	47	96	33	63
Age (months)	92.40±5.33	77.35±7.00	59.14±8.45	91.68±10.18	105.57±7.69	68.71±12.55	124.17±8.79
Body weight (kg)	4.00±0.09	4.51±0.12	4.44±0.19	4.57±0.16	3.55±0.11	3.30±0.17	3.68±0.13

Female cats had a significantly higher age compared to male cats (p=0.0013 for Gr. 1 and p=0.0001 for Gr. 2). Moreover, gonadectomized cats had a significantly older age than intact cats (p<0.0001 for both male and female cats for Gr. 1 and p<0.0001 and p=0.0002 for male and female cats for Gr. 2, respectively). Moreover, the female cats in both groups had significantly lower body weight compared to the male cats (p<0.0001), and gonadectomized cats had significantly higher body weight than intact cats (p<0.0001 for both male and female cats for Gr. 1 and p=0.0564 and p=0.0087 for male and female cats for Gr. 2, respectively).

### Radiographic lumbosacral vertebrae

Of 1365 cats, there were 959 and 406 cats in Gr. 1 and Gr. 2, respectively. In Gr. 1, the average lengths of L2, L5, and LL were 17.78±0.04 mm, 21.51±0.05 mm, and 16.38±0.06 mm, respectively. The average lengths of L2, L5, and LL in each group by sex and gonadal status are shown in Table-2. Interestingly, the lengths of L2, L5, and LL in male cats were significantly greater than those of female cats (p<0.0001). Furthermore, gonadectomy showed an effect on lumber vertebral length. Gonadectomized cats had significantly longer L2, L5, and LL than intact cats (p<0.0001 for L2 and L5 and p=0.0328 for LL in male cats and p=0.0089 and p=0.01235 for intact and spayed female cats, respectively). Similar results were detected on the LL of female cats, but no statistical significance was observed (p=0.5474).

**Table-2:** Lumbar vertebral lengths (mean±SEM; mm) of the second lumbar vertebra (L2), the fifth lumbar vertebra (L5), and the last lumbar vertebra (LL) of cats, all of the overall, normal lumbar sacral vertebrae, and non traumatic lumbar sacral lumbar abnormalities.

Lumbar vertebral lengths	Gonadal status						

	Overall	Male	Intact male	Castrated male	Female	Intact female	Spayed female
Overall cats							
L2	17.78±0.04	18.26±0.05	17.95±0.07	18.63±0.06	17.15±0.05	16.90±0.08	17.36±0.07
L5	21.51±0.05	22.08±0.07	21.67±0.09	22.57±0.07	20.74±0.06	20.45±0.09	20.99±0.07
LL	16.38±0.06	16.93±0.07	16.75±0.01	17.14±0.10	15.66±0.08	15.51±0.12	15.78±0.11
Normal vertebrae							
L2	17.75±0.05	18.25±0.06	17.94±0.08	18.62±0.08	17.07±0.06	16.81±0.09	17.32±0.08
L5	21.55±0.05	22.17±0.07	21.80±0.09	22.36±0.09	20.69±0.07	20.40±0.11	20.97±0.09
LL	16.23±0.06	16.78±0.08	16.60±0.12	16.99±0.11	15.47±0.09	15.32±0.13	15.61±0.13
Abnormal vertebrae							
L2	17.86±0.07	18.30±0.10	17.97±0.16	18.67±0.11	17.32±0.10	17.17±0.15	17.41±0.13
L5	21.40±0.09	21.84±0.13	21.33±0.20	22.44±0.13	20.86±0.11	20.59±0.19	21.02±0.14
LL	16.76±0.11	17.31±0.14	17.15±0.21	17.48±0.19	16.08±0.16	16.06±0.27	16.09±0.19
Congenital							
L2	17.95±0.11	18.25±0.14	17.90±0.20	18.74±0.16	17.46±0.15	17.20±0.22	17.64±0.20
L5	21.37±0.12	21.66±0.17	21.19±0.25	22.32±0.18	20.90±0.16	20.53±0.27	21.15±0.18
LL	17.40±0.14	17.72±0.18	17.42±0.26	18.13±0.24	16.87±0.22	16.60±0.35	17.05±0.27
Acquired							
L2	17.75±0.10	18.38±0.15	18.11±0.26	18.59±0.16	17.20±0.12	17.00±0.25	17.23±0.16
L5	21.44±0.13	22.15±0.18	21.65±0.33	22.54±0.19	20.83±0.15	20.45±0.31	20.92±0.19
LL	15.95±0.15	16.61±0.21	16.56±0.33	16.65±0.27	15.36±0.20	15.37±0.40	15.31±0.23

In Gr. 2, 226 cats were categorized to Gr. 2A (congenital abnormalities), whereas 180 cats had acquired lesions (Gr. 2B). In Gr. 2A, 207 cats had a single lesion, and 19 cats had multiple lesions. Among cats with single lesion of congenital abnormalities, there were six lumbar vertebrae (n=141), sacralizaton (n=48), lumbarization (n=16), hemivertebrae (n=1), and five lumbar vertebrae (n=1). In contrast, in Gr. 2B, 89 cats had a single lesion, and 91 cats had multiple lesions. Among cats with acquired lumbosacral vertebral abnormalities, there were bone spur (n=45), narrowing disk space (n=18), spondylosis deformans (n=17), and lumbosacral degeneration (n=9). In the single vertebral abnormality group, the number of areas found in Gr. 2, Gr. 2A, and 2B was 21/296, 2/207, and 19/89 cats, respectively, for the cranial area and 275/296, 205/207, and 70/89 cats, respectively, for the caudal area.

Between groups, Gr. 2A had a significantly younger age than Gr. 2B (p<0.0001). However, the body weight between the two groups was not statistically different (p=0.9986).

### Large bowel characterization on abdominal radiograph

**Figure-2:**
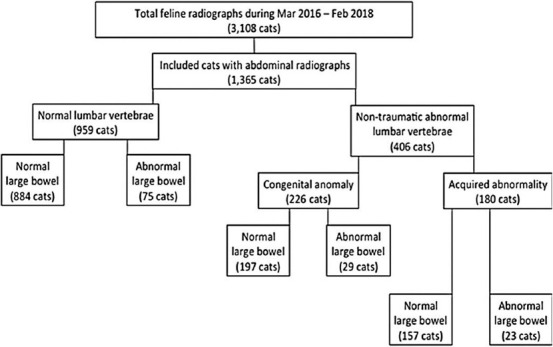
A diagram indicating the distribution number of the cats in each group, categorized by the presentation of lumbar-sacral lumbar vertebral characteristic and the presentation of large bowel dysfunction.

Applying the cutoff values to differentiate between cats with constipation and megacolon, there were 75/959 cats in Gr. 1 with large bowel dysfunction problems, 55 with constipation, and 20 with megacolon, whereas 52/406 cats in Gr. 2 had large bowel dysfunction problems, 31 with constipation, and 21 with megacolon. Cats in Gr. 2 had significantly increased risk of abnormal colon diameter than those in Gr. 1 (p=0.0057; odds ratio=1.731). Considering each subgroup, the numbers of cats affected by constipation and megacolon were 19/226 and 10/226 for Gr. 2A and 12/180 and 11/180 for Gr. 2B, respectively. Moreover, a statistical association of abnormal colon diameter of cats in Gr. 2A and 2B was found compared to that in Gr. 1 (p=0.0069; odds ratio=1.920 and p<0.0001; odds ratio=4.107) (Figure-2). Furthermore, by location, cats with a single location of lumbosacral vertebral abnormalities in the caudal area had increased risk of constipation and megalocon (38/275 cats, p=0.0044; odds ratio=1.865), while those with cranial location (2/21) did not show statistical association (p=0.6804).

## Discussion

Abdominal radiography is an efficient diagnostic tool for detecting any abnormalities of the abdominal internal organs. Among these, GI diseases, especially of the distal GI tract, were periodically detected ­clinically, for example, diarrhea, GI foreign body, intramural or extramural GI lesions, constipation, obstipation, and/or megacolon. The latter GI abnormalities could be induced by several underlying causes, such as narrowing pelvic canal and idiopathic conditions. Idiopathic constipation and/or megacolon may involve generalized dysfunction of colonic smooth muscle. The etiology is still unclear [3]. Although constipation was sometimes reported in cats, the incidence of non-obstructive constipation and its causes, such as non-traumatic lumbosacral vertebral abnormalities, is low.

This study aimed to demonstrate the incidence of non-traumatic lumbosacral vertebral abnormalities in cats and relationships of the abnormalities to large bowel dysfunction in cats observed on a retrospective model using abdominal radiographs. The statistical analysis was performed only in the normal vertebral group since nontraumatic lumbosacral vertebral abnormalities could affect the measurement of lumbar vertebral length and lead to unreliable results. The results revealed the statistical differences of lumbar vertebral length between male and female cats, which are consistent with the previous study in humans, indicating that gonadal hormones of both sexes play an important role as initiators of bone growth spurt and mineralization through complex mechanisms, such as bone resorption inhibition and stimulation of active Vitamin D3 metabolite production [24]. Androgens can also enhance the skeletal sensitivity to calcitonin in rats [25]. In addition to sex, gonadal status also has an effect on lumbar vertebral length in both male and female cats. This effect might be caused by prepubertal gonadectomy. It has been reported that prepubertal gonadectomy has an effect on bone growth by delaying the closure time of physis [26]. Despite the retrospective model indicating that the history of the gonadectomy period for each cat could not be obtained, authors assumed that most gonadectomized cats in this study might have undergone gonadectomy before the prepubertal period. Prepubertal gonadectomy can assist in population control in cats, which are a seasonal polyestrus species [27,28], and additionally reduce the risk of feline mammary gland tumors, especially in female cats [29]. This phenomenon is more clearly observed in male cats compared to female cats. The discrepancy of the effect of gonadal status on physeal closure between sexes of the cat might be caused by sex hormonal imbalance in juvenile male cats that might affect bone maturation. However, further investigations should be undertaken to clarify this.

The age range of the congenital subgroup was significantly lower than that of the acquired subgroup. This could be due to the embryologic development of the vertebrae that are closely related to spinal cord or other organ abnormalities [30]. Owners could detect abnormal clinical signs of various organs; then, cats were presented earlier to the veterinarian. In contrast, cats with acquired bone abnormalities might gradually reveal clinical signs over time, in accordance with degenerative diseases of organs. As a result, the age at which the acquired lumbosacral vertebral abnormal cats were presented to the veterinarian might be later.

Furthermore, 70.26% of cats in this study had normal bone condition, while the other 29.74% were considered otherwise. In the abnormal group, 16.55% and 13.19% of cats had congenital and acquired lumbar vertebral abnormalities, respectively. The most common congenital lumbar vertebral abnormality in this study was an abnormal number of lumbar vertebrae, which accounted for 68.12%. There were also studies on abnormal vertebral numbers found in several other species, such as ferrets and rabbits [31,32]. This abnormality may have clinical significance from the possible radiographic misinterpretation and inaccurate choice of surgical site. The most common acquired bone abnormality found in this study was ventral bone spur (50.56%). As bone spurs develop, they can create pressure on exiting spinal nerve roots and possibly result in neurologic deficits. Although the osteophytes typically do not project into the spinal canal, the possibility of spinal cord pressure must also be considered [33].

Overall, 52 (12.80%) cats with lumbar vertebral abnormalities had radiographic evidence of an abnormal large bowel, in which colon diameter was significantly larger than that of cats with normal lumbar vertebrae of 75 (7.82%) (p=0.0057). The association of vertebral abnormalities and GI disorders in cats has never been clearly described. The feline idiopathic megacolon has been attributed to primary neurologic and degenerative neuromuscular disorders, and the most frequent cause is that these vertebral abnormalities damage the lumbosacral plexus, which originates from the level of L4 through the sacrum, which may affect the autonomic function of the pelvic viscera [2,34]. Sacralization or the abnormality of last lumbar vertebrae can also result in clinical signs of lumbosacral disease, including terminal colon atony [34].

This retrospective study has some limitations. The radiographic evaluation of lumbar vertebra should be obtained from spot radiographs to avoid distortions, which could cause misinterpretation. In addition, a calibration ball should be applied for increased accuracy and to avoid variation in magnification among the measurements due to different distances between objects and films. Although this study revealed that the lumbar vertebral abnormalities may be related to constipation or megacolon in cats, the association of the abnormal bone location and the affected nerves supplying the colon should be further investigated to clarify the pathogenesis of the large bowel abnormalities. In 2005, Chang *et al*. [35] reported that the innervations of rectal detrusor and external anal sphincter in dogs are provided by the ventral roots of L7, S1, S2, and S3, which originate from the sacral spinal cord parasympathetic and somatic centers. In addition, since large bowel dysfunction, both constipation and megacolon, could be induced by other causes, such as concurrent diseases, dehydration, diet, and other medications, the complete clinical information through physical examination, including multifactorial observation, could provide evidence.

## Conclusion

This study unveiled radiographic information concerning lumbosacral vertebral abnormalities and large bowel dysfunction in cats. This information could assist veterinarians in evaluating, monitoring, and obtaining awareness of constipation or megacolon problems in cats with incidental findings of vertebral abnormalities on abdominal radiographs. Although the clinical signs are not immediately apparent, they continuously progress. Veterinary practitioners should perform an early, thorough diagnostic workup to define the potential of disease progression and provide proper advice to cat owners for prevention and control of large bowel functions. Moreover, a neurological examination should also be performed to confirm problems in cases diagnosed with both intestinal and lumbosacral vertebral abnormalities. Therefore, the information found on abdominal radiographs of cats, both distal GI and vertebral lesion, could assist the owners and veterinarians to undertstand the cat’s condition and provide proper management to improve the quality of their lives.

## Authors’ Contributions

CT and NC: Study conception and design. KK, KB, NT, and WS: Acquisition of data. CT, KK, KB, NT, WS, and NC: Analysis and interpretation of data. CT, KK, KB, NT, WS, and NC: Drafting of manuscript. CT, KK, KB, NT, WS, and NC: Critical revision. All authors have read and approved the final manuscript.
